# Glycyrol Suppresses Collagen-Induced Arthritis by Regulating Autoimmune and Inflammatory Responses

**DOI:** 10.1371/journal.pone.0098137

**Published:** 2014-07-18

**Authors:** Yanxia Fu, Hailing Zhou, Shuangyan Wang, Qun Wei

**Affiliations:** Department of Biochemistry and Molecular Biology, Beijing Normal University, Gene Engineering and Biotechnology Beijing Key Laboratory, Beijing, P. R. of China; INSERM-Université Paris-Sud, France

## Abstract

Glycyrol is a natural compound extracted from *Glycyrrhiza uralensis*, first reported by us to be a new immunosuppressant. Here, we demonstrate its beneficial effect in collagen-induced arthritis (CIA) in mice, a model for rheumatoid arthritis (RA) in man, and we document the underlying mechanisms. Peroral administration of glycyrol significantly reduced clinical scores, alleviated cartilage and bone erosion and reduced levels of serum inflammatory cytokines. Glycyrol also decreased delayed-type hypersensitivity, improved carbon clearance and reduced acetic acid-induced capillary permeability. Furthermore, glycyrol decreased NF-κB and NFAT transcriptional activities and inhibited IL-2 expression. The therapeutic effect of glycyrol was associated with down-regulation of both autoimmune and inflammatory reactions. In addition, we demonstrated that glycyrol has minimal acute toxicity in mice. Therefore, we propose that glycyrol may hold promise for future treatment of RA.

## Introduction

Rheumatoid arthritis (RA) is an autoimmune disease characterized by synovial hyperplasia and inflammatory cell recruitment eventually leading to destruction of cartilage and bone [1], [2]. It afflicts approximately 1% of the worldwide population [3]. Despite intensive research, the exact etiology of RA remains obscure, and a satisfactory cure remains elusive [4]. Nonsteroidal anti-inflammatory drugs are widely used, but carry the risk of gastrointestinal toxicity [5]. Cytokine antagonists likewise decrease inflammation and joint destruction but interfere with immune defense. Additionally, these inhibitors must be injected parenterally, thereby increasing the risk of infections [6], [7]. Immunosuppressive agents, such as cyclosporin A (CsA) and tacrolimus (FK506), which have been used widely in organ transplantation and autoimmune diseases, are also useful [8], [9]. However, their use is plagued by severe side effects, notably nephrotoxicity and hepatotoxicity [10], [11], [12]. Therefore, a need is felt to develop perorally administered drugs with minimal toxicity to reduce inflammation responses and inhibit autoimmune responses. Due to the side effects of anti-arthritis drugs, an increasing proportion of patients with arthritis are resorting to complementary and alternative medicine (CAM) for their health needs. The most popular CAM treatments for inflammatory and immune disorders are found among natural plant products [13].

Our group has focused on the immunosuppressant screening of natural products with calcineurin (CN) as the target enzyme. In our previous work, we screened glycyrol from *Glycyrrhiza uralensis*, and showed that it is an immunosuppressive agent; as its intraperitoneal injection could prolong graft survival in allogeneic skin transplantation [14]. It has been reported that CN plays crucial roles in the autoimmune and inflammatory responses and that the activity of CN correlates well with the severity of arthritis in an animal model of RA. Therefore, we used collagen-induced arthritis (CIA) in mice to study the potential beneficial effect of glycyrol on the pathogenesis of arthritis. Tests for delayed type hypersensitivity (DTH) and carbon clearance were used evaluate the influenceon the immunoregulation of mice, and the acetic acid-induced capillary permeability test was used to detect the influence of glycyrol on inflammatory reactions. We show that treatment with glycyrol has great benefits at both the clinical and the pathological levels and that the therapeutic effect is associated with the down-regulation of both inflammatory and autoimmune reactions.

## Materials and Methods

### 2.1 Ethics statement

All animal experimental procedures were approved by the Animal Ethics Committee of Beijing Normal University and were conducted in strict accordance with institutional guidelines. All efforts were made to minimize the number of animals used and to reduce their suffering.

### 2.2 Materials

Glycyrol (C_21_H_18_O_6_, MW:366.4, purity>95%) was synthesized by Shuangyan Wang according to the published procedure [15]. For peroral administration to mice, the compound was dissolved in peanut oil containing 1% DMSO and infused into the oesophagus through a canula. Bovine type II collagen and complete Freund's adjuvant were purchased from Chondrex (USA). Specific-pathogen free male DBA/1J mice were acquired from the National Resource Center for Mutant Mice Model Animal Research Center of Nanjing University (Nanjing, China). Specific pathogen-free male BALB/c mice (6–8 weeks of age) were purchased from the Vital River Laboratories (Beijing, China). ELISA kits were purchased from Hin Bo Sheng Biological Technology Co, Ltd. (Beijing, China). TRIzol reagent was purchased from Bio Teke corporation (Beijing, China). p-IkBα antibodies were purchased from Cell Signaling Technology (USA). CsA was purchased from Shanghai Pureone Biotechnology (Shanghai,China). Dual-Luciferase Reporter Kits were purchased from Promega (USA).

### 2.3 Induction of CIA

CIA was induced as previously described [16]. Briefly, male DBA/1J mice were injected intradermally at the base of the tail with 100 µl of a 1/1 (v/v) emulsion of 0.1 M acetic acid containing 100 µg of bovine type II collagen (Chondrex) and complete Freund's adjuvant containing 100 µg of heat-killed *Mycobacterium tuberculosis.* The day of the first immunization was defined as day 0. Three weeks after the primary immunization, the mice were boosted with 100 µg of collagen II in Freund's incomplete adjuvant via the same route.

### 2.4 Clinical assessment of arthritis

The severity of arthritis was quantified every other day by visual inspection, as described previously [17]. The clinical severity of arthritis in the paws was evaluated double-blindly using the following semi-quantitative scoring system: (0) no evidence of erythema and swelling, (1) swelling and erythema of the digit, (2) mild erythema of the limb, (3) gross erythema and swelling of the digit, and (4) ankylosis of the limb or gross swelling deformity and inability to use the limb. The clinical score for each mouse was the sum of the four paw scores, which results in a maximum score of 16. On the final day of the experiments, all mice were anesthetized with pentobarbital, and their blood was collected by cardiac puncture. In addition, their hind paws and knee joints were harvested for histological examination.

### 2.5 Histopathologic Analysis

Removed hind paws were fixed in 4% phosphate-buffered paraformaldehyde solution, decalcified in 30% formic acid and citric acid, and then embedded in paraffin. The tissues were longitudinally cut into 5-µm serial sections and stained with hematoxylin and eosin (H&E). The histopathological changes in the joints were examined under light microscopy and scored with a value ranging from 0 to 3, where 0 = normal, 1 = mild synovitis or slight cartilage erosion, 2 = moderate synovitis and cartilage erosion, and 3 = erosion of the cartilage or bone destruction [18].

### 2.6 DNFB-induced DTH assay

DTH assays were conducted as previously described [19]. The fur was removed (2 cm*2 cm) to expose the abdomen. The mice were initially sensitized by uniformly painting 50 µL of 1% 2,4-dinitrofluorobenzene (DNFB) dissolved in acetone and peanut oil (1∶1) on their shaved abdomens on days 1 and 2. The mice in the negative control group were painted with 50 µL of solvent without DNFB. Three days after the second sensitization (day 5), all mice were treated with 10 µL of 1% DNFB on both sides of their right ear, and the left ears were treated with solvent alone. The mice were killed by cervical dislocation 24 h after treatment, and the spleen and thymus were immediately removed and weighed. The spleen and thymus indexes were expressed as spleen weight (mg) per 10 g of body weight and the thymus weight (mg) per 10 g of body weight, respectively. The ear swelling was expressed as the difference between the weights of the left and right ear patches obtained from 8-mm punches 24 h after the third sensitization.

### 2.7 Carbon particle clearance test

A carbon clearance experiment [20] was used to test the effect of glycyrol on the phagocytic activity of macrophages. Indian ink was centrifuged for 10 min at 3000 rpm; the supernatant was diluted 1∶4 with sterile physiological saline and then injected into the tail vein of BALB/c mice (0.1 ml per 10 g body weight) 30 min after the last peroral administration. 2 min (T1) and 10 min (T2) after the injection of the ink, 20 µL of blood obtained from the retro-orbital venous plexus was added into 2 mL of 0.1% solution of Na_2_CO_3_ to lyse the red cells. The absorbances at 675 nm of the blood collected at 2(C1) and 10 min (C2) were measured, and the absorbance of the blood from the normal control group was set to zero. The mice were sacrificed by cervical dislocation, and the liver and the spleen weights were measured. The clearance index (K) and the phagocytic index (α) were calculated using the following equations: k =  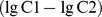/(T2-T1) and α = 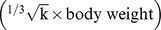/(liver weight +spleen weight), respectively.

### 2.8 Acute toxicity test

The mice were subjected to an acute toxicity test using the fixed-dose procedure, which is a sequential testing scheme that was proposed by the British Toxicology Society in1984 as an alternative for the assessment of acute peroral toxicity via estimation of the Lethal Dose 50 (LD_50_). The procedure is incorporated into the European Community Directive guidelines as the acute peroral toxicity test [21]. Briefly, an initial dose of 5, 50, 500, or 2000 mg per kg of body weight can be selected to evaluate the toxicity of the substance being investigated. Either 5 or 2000 mg per kg can serve as the starting dose. The procedure is terminated when either toxicity or death is observed.

### 2.9 Acetic acid-induced capillary permeability test

0.5% Evan's blue solution in saline (0.1 mL per 10 g of body weight) was injected into the tail vein 30 min after the last peroral administration. After 20 min, 0.6% acetic acid in saline (0.1 mL per 10 g of body weight) was injected into the peritoneal cavity to increase the capillary permeability. 20 minutes after the acetic acid injection, the mice were sacrificed by cervical dislocation, and saline (5 mL per mouse) was injected into the abdominal cavity. After a gentle knead of the abdominal region, the peritoneal fluid was collected and centrifuged at 3000 rpm for 10 min. The supernatants were collected and color was measured photometrically as absorbance at 590 nm wavelength [22].

### 2.10 Western blot analysis of IκB-α in Jurkat cells

The phosphorylation of IκB-α was induced by incubating 1*10^6^ Jurkat cells with 100 ng/ml phorbol 12-myristate 13-acetate (PMA) plus 1 µM Ionomycin (Ion) or a combination of glycyrol and PMA plus Ion. After 24 h, the cells were harvested by centrifugation and washed twice with phosphate-buffered saline. The cells were lysed on ice for 20 min,the cell-free supernatant was collected for Western blot analysis. The protein quantification was performed using abicinchoninic acid protein quantitation kit. The samples were separated using discontinuous SDS-PAGE. The separated proteins were transferred to polyvinylidene fluoride membranes and then soaked in 5% non-fat dried milk in Tris-buffered saline for 1 h. The membrane was incubated with the primary antibody at room temperature for 1 h, incubated with a biotinylated secondary antibody for 1 h, and then developed with peroxidase-conjugated streptavidin for 1 h. The specific bands were visualized using an electrochemiluminescence kit. Reactions were performed in triplicate.

### 2.11 Dual-luciferase reporter gene assay

The NFAT and NF-κB transcriptional activity was investigated using two reporter gene assay systems. RAW 264.7 macrophage cells plated in 24-well plates were co-transfected with the p-firefly luciferase (FL)-NFAT or NF-κB plasmid and the p-renilla luciferase (RL)-SV40 endogenous control plasmid, respectively. 24 hours after transfection, the cells were incubated with PMA plus Ion in the presence or absence of glycyrol. The NFAT and NF-κB transcriptional activity was measured using Dual-Luciferase Reporter Kit according to the manufacturer's protocol. Reactions were performed in triplicate.

### 2.12 Real-time quantitative PCR for IL-2

The RNA was extracted from the RAW264.7 macrophages using the TRIzol reagent as described by the manufacturer. Real-time quantitative PCR was performed to detect the transcriptional level of IL-2. The primer sequences for IL-2 were 5′-CCTGAGCAGGATGGA GAATTACA-3′ (forward) and 5′-TCCAGAACATGCCGCAGAG -3′ (reverse).β-actin:5′-AGA GGGAAATCGTGCGTGAC-3′(forward), and5-’CAATAGTGATGACCTGGCCGT-3′ (reverse). β-actin was used as an endogenous control gene. The real-time quantitative PCR was performed on an Applied Biosystems 7500 Real-Time PCR System using SYBR Green Dye. The reactions were performed in triplicate and the results are shown as the means ± SD.

### 2.13 Statistical analysis

Statistical comparisons were performed using one-way ANOVA and paired samples were compared by the Student's t-test using SPSS 11.0 software. The GraphPad Prism5 software was employed to calculate and draw plots. Values of P< 0.05 were considered as indicative of statistical significance.

## Results

### 3.1 Glycyrol attenuates collagen-induced arthritis

**Figure 1:**
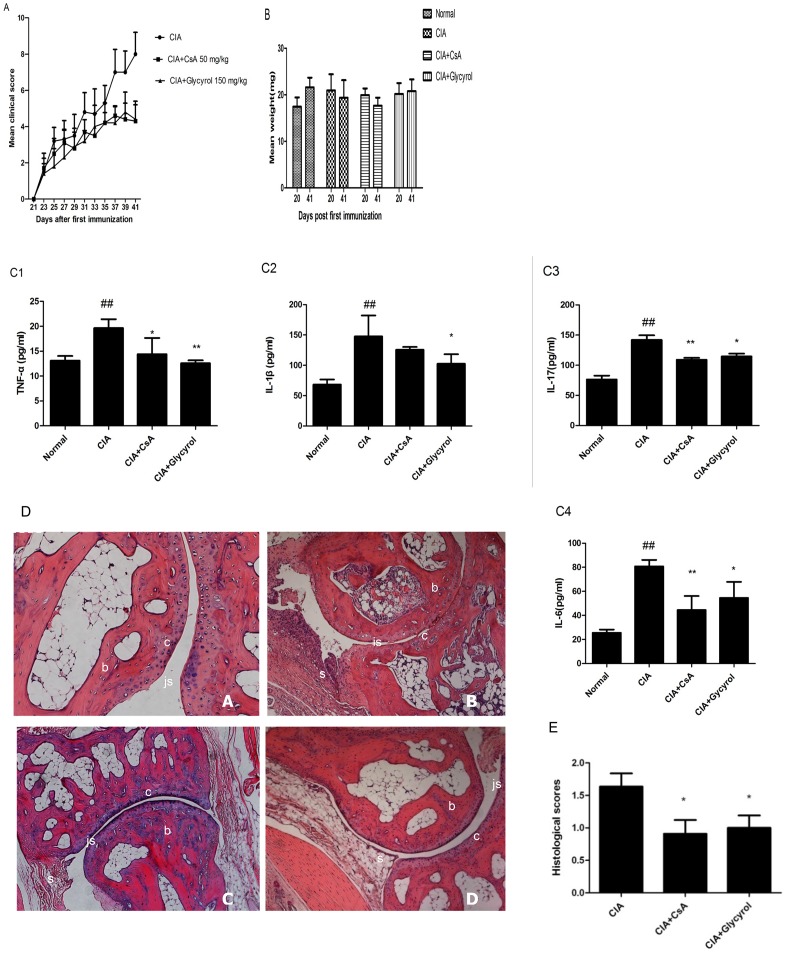
Glycyrol attenuates collagen-induced arthritis. CIA was induced by intradermal injection collagen II in Freund's adjuvant as described in Materials and Methods. A – C. Glycyrol and CsA were administered perorally every day from days 21 to 42. A: Time-dependent mean clinical scores (±SD) of four paws; B: Body weight changes (mean±SD); C1: TNF-α levels in serum on day 42; C2: IL-1β levels in serum on day 42; C3: IL-17 levels in serum on day 42; C4: IL-6 in serum on day 42. Data points are means ± SD of 7 mice. Differences between groups were tested by Student's t-test. # P<0.05 for comparison with Normal group, *P<0.05 for comparison with CIA group. D. Histopathologic features of representative ankle joints of male DBA/1J mice obtained on day 42 and stained with H&E (100x original magnification). “A” represents non-immunized mice showing normal articular cartilage, absence of infiltrate in the synovium and open joint space, “B” indicates CIA mice showing marked infiltration of inflammatory cells, narrow joint space with synovial hyperplasia, and “C” and “D” represent CIA mice treated with CsA or glycyrol, respectively, showing less inflammatory cell infiltration, well-preserved joint spaces, and minimal synovial hyperplasia. “b” denotes bone tissue, whereas “c”, “s”, and “js” indicate cartilage, synovium, and joint space, respectively. E. Quantification of histological changes. Data represent means ±SD from 7 mice. *P<0.05 for comparison with CIA group.

Mice were immunized to induce CIA (see Materials and Methods). After the secondary immunization, they were randomly divided into four groups of 7: (1) normal non-immunized group, (2) CIA group, (3) CIA plus CsA group (50 mg per kg), and (4) CIA plus glycyrol group (150 mg per kg). Glycyrol and CsA were administered perorally every day from days 21 to 42. On the final day, blood samples were collected via cardiac punctures. The contents of TNF-α, IL-1β, IL-6, and IL-17 in the serum were measured using ELISA kits according to the manufacturers' instructions. The measurements were done in triplicate. The experimental results show that both the CsA and the glycyrol treatments reduced the mean arthritis scores (Figure 1A), and reduced the contents of IL-1β, TNF-α, IL-6, and IL-17(Figure 1C) in the serum. By the final day of the experiments, the weights of the normal and the glycyrol-treated mice had increased, whereas those of the mice in the CIA and CsA groups had decreased (Figure 1B). The histopathological changes in the joints were examined under light microscopy and scored with a value ranging from 0 to 3 based on the observed synovitis and cartilage/bone destruction. As the analysis of the histological changes (Figures 1D and 1E) suggests, there was obvious evidence of synovial hyperplasia, joint space narrowing, and bone and cartilage erosion in the joints of the mice in the CIA group, whereas these changes were scarcely observed in the mice in the glycyrol-treated group.

### 3.2 Inhibitory effect of glycyrol on immunoregulation

**Figure 2:**
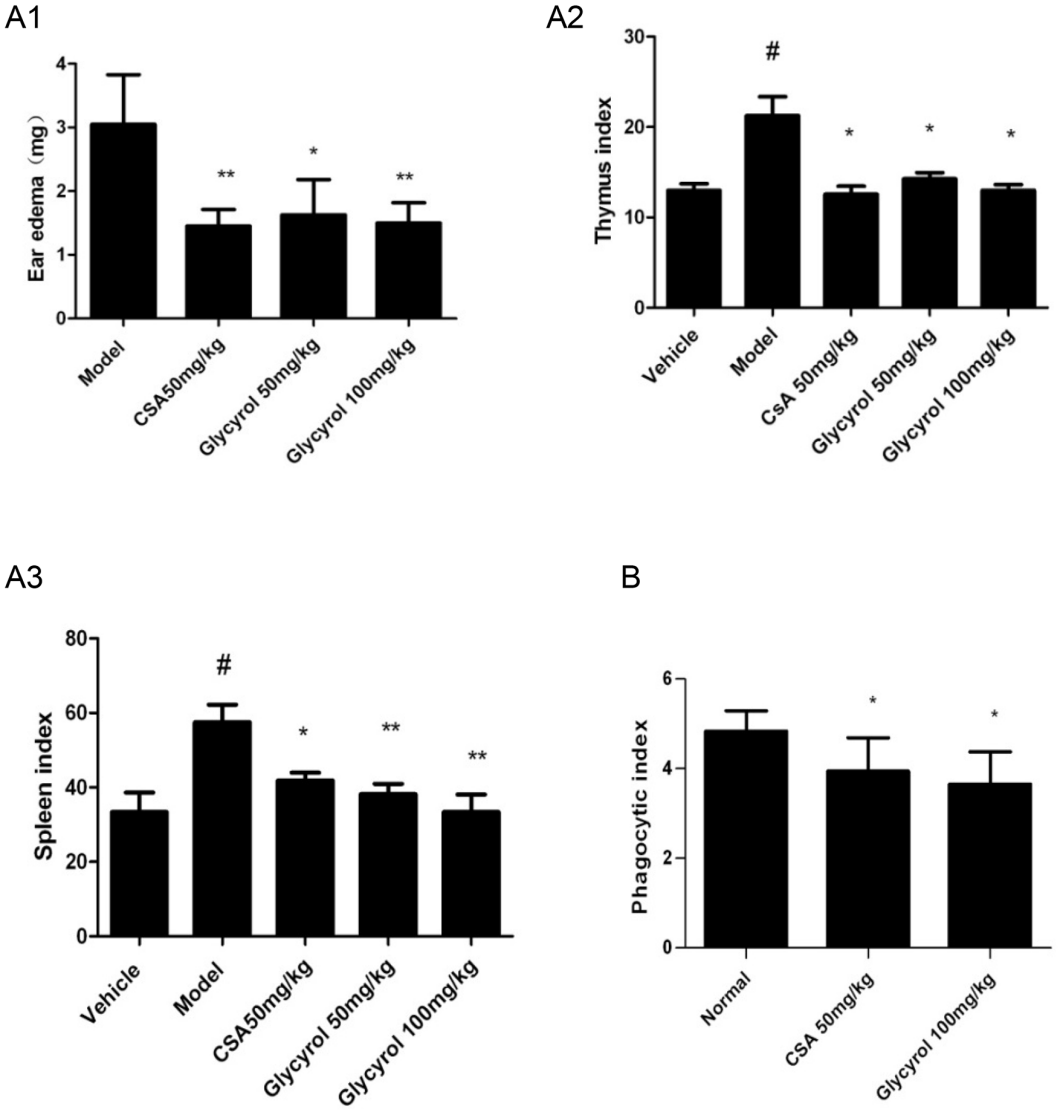
Effect of the peroral administration of glycyrol on immunoregulation *in vivo.* DNFB-induced DTH reaction (panel A) and carbon particle clearance test (panel B) conducted on mice given glycyrol (50 or 100 mg/kg) or CsA (50 mg/kg). The mice were initially sensitized by 50 µL of 1% DNFB on abdomens 30 min after drug administration on days 1 and 2. On day 5, 30 min after drug administration, mice were treated with 10 µL of 1% DNFB on both sides of their right ear. The mice were killed by cervical dislocation 24 h after the third sensitization. A1: ear swelling; A2: thymus; A3: spleen; B: phagocytic index. Data are shown as means ±SD from 8 mice. Differences between groups were tested by Student's t-test. # P<0.05 for comparison with Vehicle group, *P<0.05 for comparison with Model group.

The DTH reaction and the carbon particle clearance test were employed to evaluate the influence of glycyrol on immunoregulation. Our previous work had demonstrated that mice injected intraperitoneally with glycyrol showed significantly decreased DTH and exhibited prolonged graft survival in allogeneic skin transplantation. To adapt to the characteristics of arthritis patients, who take drug over a long period of time, we decided to test peroral administration of glycyrol. For the DTH test, 40 male BALB/c mice were randomly divided into five groups of 8: a negative control group, a model group, a CsA group, and two glycyrol groups. The mice in the CsA group received peroral doses of 50 mg/kg CsA daily for six days, and the mice in the glycyrol groups received 50 or 100 mg/kg peroral glycyrol daily for six days. The mice were initially sensitized by DNFB on abdomens 30 min after drug administration on days 1 and 2, then on both sides of their right ear 30 min after drug administration on day 5. Figure 2A shows that the administration of glycyrol has a dose-dependent effect on DNFB-induced DTH because the ear swelling was significantly lower in the glycyrol-treated groups than in the model group. The results indicate that both glycyrol and CsA inhibit the increase in the spleen and thymus indexes induced by DNFB and that the inhibitory effect of glycyrol on the spleen index is better than that on the thymus index. Furthermore, the inhibitory effect of glycyrol on the spleen index is better than that of CsA (Figure 2A).

For the carbon clearance test, 24 male BALB/c mice were randomly divided into 3 groups of 8: a normal group, a CsA group (50 mg/kg), and a glycyrol group (100 mg/kg). The mice in CsA or glycyrol group were administered perorally every day for three days. 30 min after the last dose, indian ink diluted with sterile physiological saline was injected into the caudal vein of every mouse. Figure 2B shows that both glycyrol and CsA reduced phagocytosis by monocytes.

### 3.3 Acute toxicity of glycyrol in mice

In the European Community, the estimated LD_50_ is used mainly to place a substance under investigation into one of four categories. The substance is classified as ‘very toxic’ if the estimated LD_50_ is less than 25 mg per kg body weight, as ‘toxic’ if it is between 25 and 200 mg per kg body weight, and as ‘harmful’ if it is between 200 and 2000 mg per kg body weight. Substances with an estimated LD_50_ larger than 2000 mg per kg body weight are termed ‘unclassified’ [23].

Fourteen male BALB/c mice were divided into two groups of 7. Glycyrol was administered perorally at a single dose of 500 mg per kg to one group and at a dose of 2000 mg per kg to the other group. The mice were then observed over a period of two weeks to assess any changes in reactivity, gait, motor activity, respiration rate, and especially death. Mice given 2000 mg/kg body weight (n = 7) glycyrol did not show any difference in their gross general behavior; no death or obvious weight changes were observed over the 14 days that the mice were observed. Thus, the LD_50_ of glycyrol is more than 2000 mg/kg, which indicates that glycyrol can be categorized as ‘unclassified.’

To further investigate the effect of glycyrol on the function of the liver and the kidney of animals, 21 male BALB/c mice were randomly divided into three groups of 7: a control group, a CsA group, and a glycyrol group. The dosage schedule was: 500 mg/kg perorally on day 0 and 100 mg/kg on days 1 and 2. The mice in the control group received an equivalent amount of the solvent. On day four, the mice were anesthetized with pentobarbital and blood was collected to determine the levels of glutamic-pyruvic transaminase, glutamic-oxaloacetic transaminase, total bilirubin, urea nitrogen, and creatinine in the serum.

**Figure 3:**
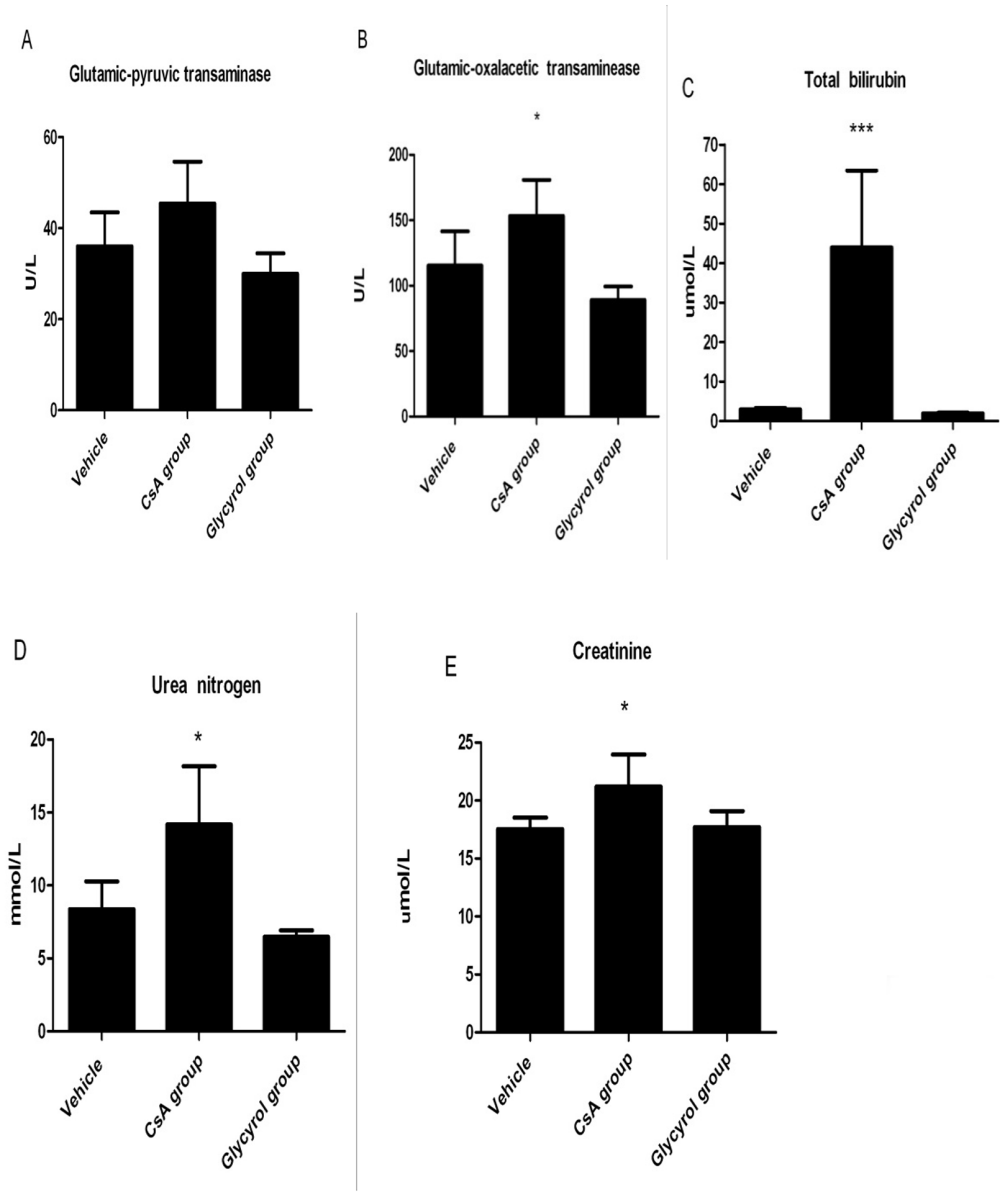
Acute toxicity of glycyrol in mice. Biochemistry in serum of mice treated with CsA or glycyrol. Dosage schedule: 500 mg/kg on day 0, 100 mg/kg on days 1 and 2; blood sampling on day 4. A: Glutamic-pyruvic transaminase; B: Glutamic-oxaloacetic transaminase; C: Total bilirubin; D: Urea nitrogen; E: Serum creatinine. Bars represent means ±SD from 7 mice. *P<0.05, **P<0.01 and ***P<0.001 for comparison with Vehicle group.

Tests on the liver and kidney function showed blood biochemistry differences between the CsA-treated and the control groups. Treatment with CsA increased the blood biochemistry characteristics of the liver and the kidney, whereas minimal changes were observed in the glycyrol group (Figure 3). Thus, glycyrol had fewer adverse effects than CsA in our experimental animals.

### 3.4 Effects of glycyrol on acetic acid-induced capillary permeability

**Figure 4:**
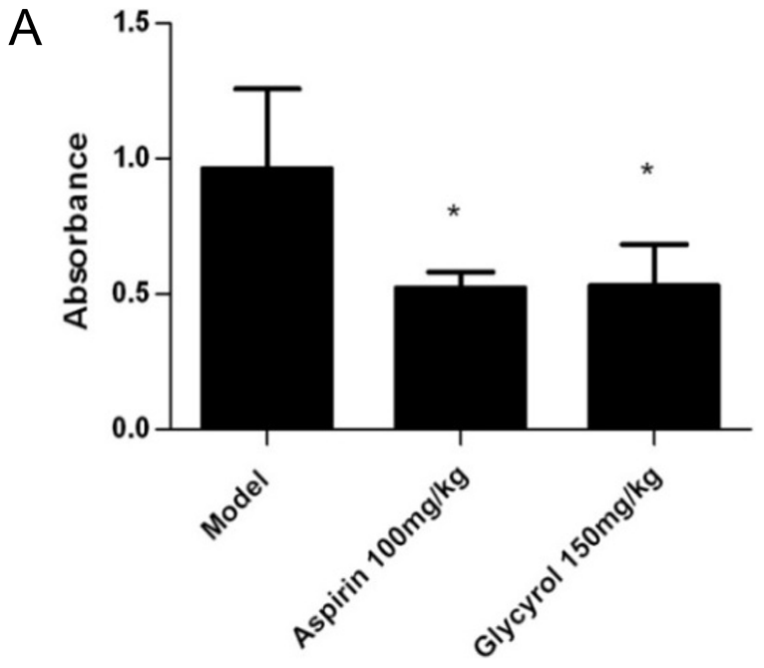
The action of glycyrol on acute inflammation *in vivo.* Acetic acid-induced capillary permeability test. Mice were given 150 mg/kg glycyrol perorally for 5 consecutive days before the acute inflammatory test. 0.5% Evan's blue was injected into the tail vein 30 min after the last peroral administration. Permeability scored by Evans Blue concentration in peritoneal fluid (light absorbance at 590 nm). Columns represent means ±SD from 8 mice. Differences between groups were tested by Student's t-test. *P<0.05 for comparison with Model group.

Acetic acid-induced capillary permeability was used to evaluate the action of glycyrol on inflammation *in vivo*. 24 male BALB/c mice were randomly divided into 3 groups of 8: control group, aspirin group, and glycyrol group. Peroral treatment with glycyrol or aspirin was administered on five consecutive days before inducing the capillary permeability test. The mice in the aspirin group received peroral doses of 100 mg/kg aspirin daily for 5 days, and the mice in the glycyrol groups received 150 mg/kg peroral glycyrol daily for five days. Figure 4 shows that glycyrol substantially reduces the increase in capillary permeability stimulated by acetic acid.

### 3.5 Glycyrol inhibits the activity of NF-κB

**Figure 5:**
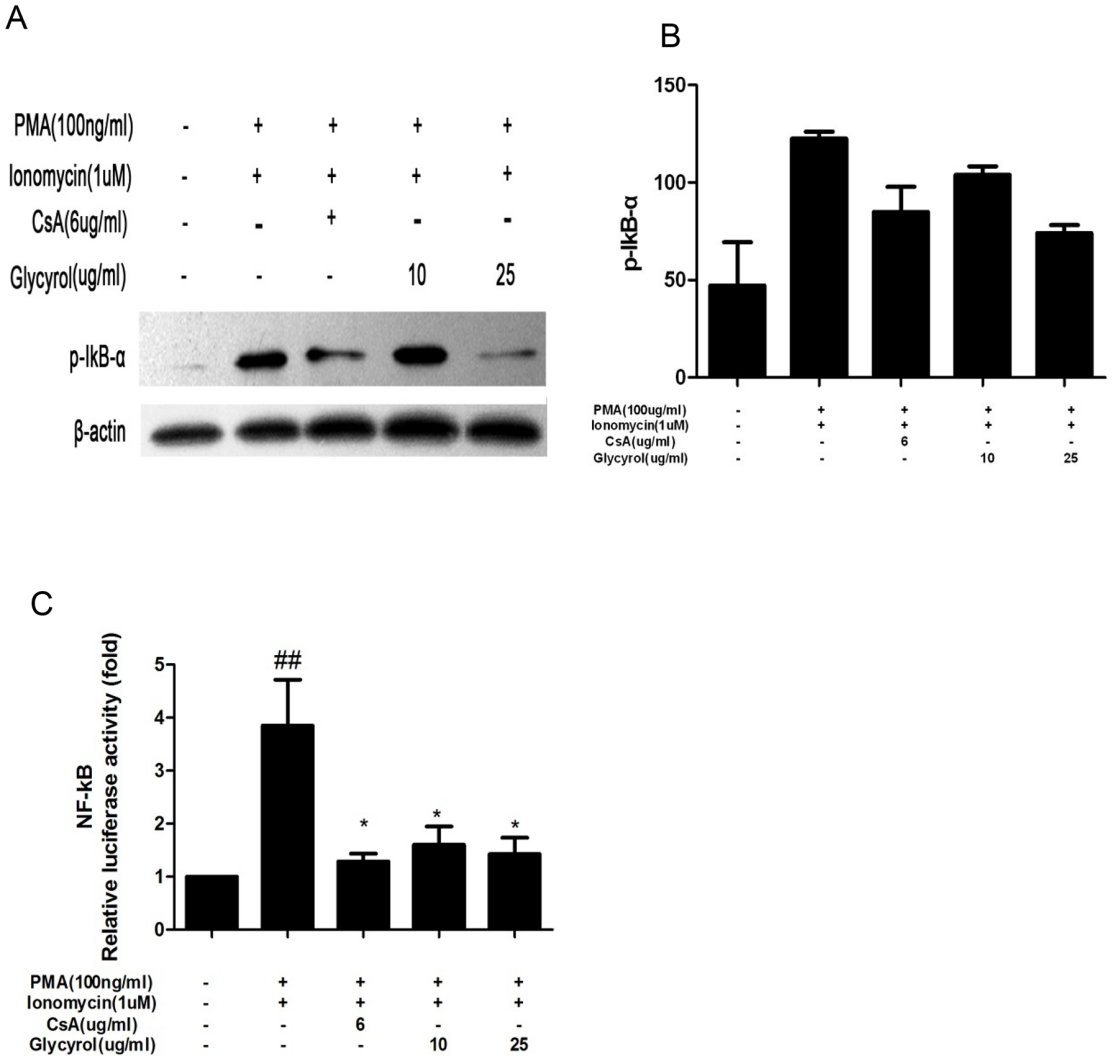
Glycyrol inhibits expression of p-IκB and NF-κB. A & B: Jurkat cells were stimulated with PMA plus Ion or a combination of indicated concentrations glycyrol or CsA and PMA plus Ion for 24 h to induce IκB-α expression. A: Western blot picture of p-IκB-α; B: Quantification of Western blot. C: RAW 264.7 cells were induced with PMA plus Ion or a combination of indicated concentrations glycyrol or CsA and PMA plus Ion for 24 h to induce NF-κB expression, and measured by dual-reporter gene assay system. Assays were performed in triplicate. Data represent means ±SD over three independent experiments. # P<0.05 for comparison with naïve group, *P<0.05 for comparison with PMA plus Ion-stimulated group.

IκB is an endogenous inhibitor of the transcription factor nuclear factor kappa B (NF-κB) which, upon phosphorylation by IκB kinase, is rapidly degraded, and this allows translocation of NF-κB to the nucleus [24]. NF-κB plays an important role in the inflammatory response. Hence, we tested the effects of glycyrol on expression of IκB-α in Jurkat cells and on NF-κB expression in RAW 264.7 cells. Cells were stimulated with PMA plus Ion or a combination of different doses glycyrol (as indicated in Figure 5) and PMA plus Ion for 24 h. Cells incubated only with plain medium or PMA plus Ion served as negative and positive controls, respectively. As shown in Figure 5, the phosphorylation of p-IκB and the transcriptional activity of NF-κB increased after stimulation with PMA plus Ion, increments that were partially prevented by simultaneous treatment with glycyrol or CsA.

### 3.6 Glycyrol inhibits NFAT activity and IL-2 transcription

**Figure 6:**
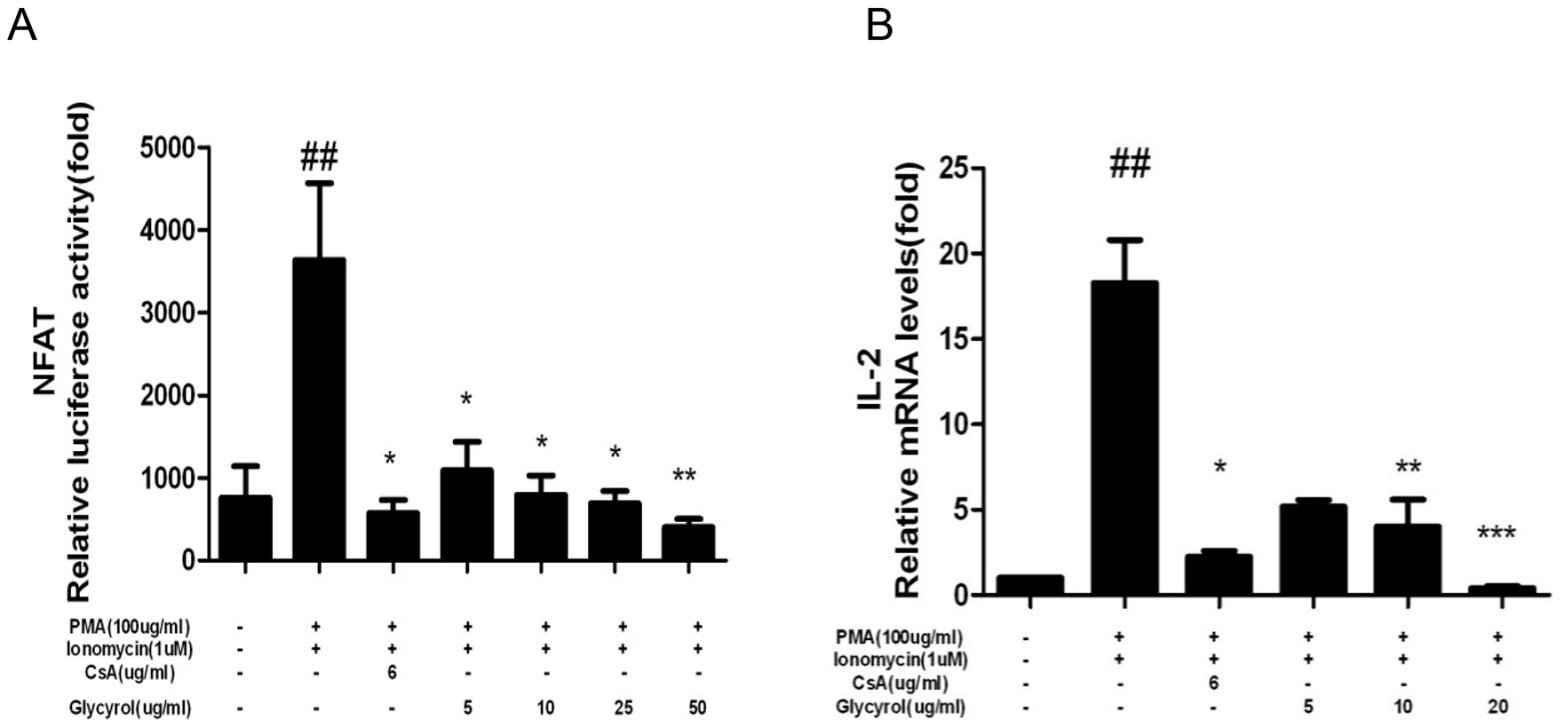
Glycyrol inhibits transcription of NFAT and IL-2genes. RAW 264.7 cells were stimulated with PMA plus Ion or a combination of indicated concentrations glycyrol or CsA and PMA plus Ion for 24-2 gene transcription. Panel A: NFAT transcription was measured using the dual-reporter gene assay system; Panel B: IL-2 gene transcription was detected by Real-time quantitative PCR. Assays were performed in triplicate. Data represent means ± SD over three independent experiments. Statistical significance was calculated using one-way analysis of variance (ANOVA) and Bonferroni post-test. # P<0.05 for comparison with naïve group, *P<0.05 for comparison with PMA plus Ion-stimulated group.

CsA displays its immunosuppressive effect by inhibiting the NFAT/IL-2 signaling pathway. To determine whether glycyrol influences the NFAT/IL-2 signaling pathway, two reporter gene assays and real-time quantitative PCR were employed to test NFAT activity and IL-2 transcription, in RAW 264.7 macrophages, respectively. Cells were stimulated with PMA plus Ion or a combination of glycyrol or CsA and PMA plus Ion for 24 h. The data in Figure 6 suggest that glycyrol dose-dependently inhibits both NFAT and IL-2.

## Discussion

We proved in this study that perorally administered glycyrol is effective in slowing down CIA as it lowered the clinical (Figure 1A) and pathological scores (Figure 1E), reduced blood levels of inflammatory cytokines (Figure 1C), and prevented joint damage (Figure 1D). We also present evidence showing that this therapeutic effect of glycyrol on arthritis is associated with a reduction in the two deleterious components of the disease, namely the autoimmune and the inflammatory responses. CN plays crucial roles in both these responses [25]. It acts mainly on T-lymphocytes and other immune cells, particularly via the CN-NFAT signaling pathway. Activated CN dephosphorylates NFAT, leading to its nuclear localization and the activation of the expression of its target genes, such as IL-2 [26], [27]. Both DTH and CIA have been identified as Th1-mediated diseases [28]. Peroral administration of glycyrol significantly inhibited CIA (Figure 1) and DNFB-induced DTH (Figure 2A), demonstrating the suppressive effect of glycyrol on T-cell-dependent immune responses. In addition, peroral administration of glycyrol was found to inhibit the phagocytosis of macrophages (Figure 2B) in the carbon clearance experiment, which suggests that glycyrol can also adjust inherent immunity.

It has been reported that the pathogenesis process of rheumatoid arthritis is mainly driven by nuclear transcription factors, such as NF-κB, which is a transcription factor that plays an important role in regulating the expression of many genes involved in immune responses, including the cytokines TNF-α, IL-1β, MMP-1, and MMP-3, all of which are closely involved in the pathogenesis of RA. CsA, which inhibit RA, hampers the activity of NF-κB and NFAT [9]. Figures 5 and 6 show that, similar to CsA, glycyrol can inhibit the activation of NF-κB and NFAT *in vitro*.

Although the precise mechanism of RA is unknown, it is clear that cytokines play a key role in the inflammation processes of RA [29]. The monocyte/macrophage product TNF-α plays a central role in joint inflammation because TNF-α is at the top of a pro-inflammatory cytokine cascade [30], [31]. IL-17 is a T cell-derived cytokine that leads to angiogenesis, joint inflammation, cartilage destruction, and bone erosion in RA [32], [33]. In several animal models of arthritis, the inhibition of IL-17 has been found to limit inflammation and joint erosion [34]. Glycyrol treatment reduced levels of TNF-α, IL-17, IL-6, and IL-1β (Figure 1C) in the serum of CIA. Furthermore, it decreased the MMPs and NO contents in the of primary and inhibited the impression of inducible nitric oxidase synthase (iNOS) and cyclooxygenase-2 (COX-2) (data not shown). Thus, it appears that glycyrol attenuates CIA through its influence on the inflammatory response.

As a whole, the therapeutic effect of glycyrol was associated with down-regulation of both the inflammatory and the autoimmune reactions. It is well established that there are some interactions between inflammatory factors and immune cells. Because NF-κB plays a key role in both the inflammation and the autoimmune responses, there are many intersections between the autoimmune and the inflammation reactions. Thus, it is difficult to precisely state how much of the RA pathogenesis is autoimmune and how much is due to inflammatory reactions. However, the effective dose in DTH and in the carbon clearance test was100 mg per kg of body weight, whereas the effective dose in the acetic acid-induced capillary permeability test was150 mg per kg of body weight (P<0.05). Thus, glycyrol maybe more powerful as an immunosuppressant than as an anti-inflammatory agent.

In this study, the acute toxicity of glycyrol was tested in mice over a period of 14 days. No mice died or exhibited behavioral changes during this period, even in the high-dose group (2000 mg per kg), which indicates that the median lethal dose (LD_50_) of glycyrol is greater than 2000 mg per kg. Our previous work on the maximum dose tolerance in mice proved that intraperitoneal administration of 400 mg/kg glycyrol three times a day also did not cause death or behavioral changes. Therefore, we can conclude that glycyrol has no or minimal toxicity. Because RA is a chronic autoimmune disease, which requires drug treatment for several months, a higher dose was employed to further test whether glycyrol impacts the liver and kidney functions. In this study, we found that the levels of total bilirubin, glutamic-pyruvic transaminase, glutamic-oxaloacetic transaminase, urea nitrogen, and creatinine (Figures 3) were increased in mice administered large peroral doses of CsA but not in mice treated with glycyrol. Overall, the results show that glycyrol has fewer adverse effects than CsA on experimental animals.

From the prescription of the medication Sandimmune (Cyclosporine), we have learned that the initial oral dosage for transplantation is 14 to 18 mg/kg in most clinical trials. The usual oral dose for autoimmune disease is 5 mg/kg, and this dose must be taken for three to six months according to the instructions. For CIA, a peroral dose of 50 mg/kg is a moderate dose for mice because this dosage is equivalent to 4 mg/kg for humans, as determined through a dose conversion using experimental pharmacological methods. In the 20-day period during which the drug was taken after the second immunization, the weight of the mice in the CsA-treated (50 mg per kg) group decreased by 11% and that of the mice in the CIA group decreased by 7%, whereas the weight of the mice in the glycyrol-treated (150 mg/kg) group increased by 2%. This result may be further proof that glycyrol is less toxic than CsA. Therefore, we have reason to expect that it will become a potential candidate drug in the future.
